# Impact of multiple small and persistent threats on extinction risk

**DOI:** 10.1111/cobi.13901

**Published:** 2022-05-05

**Authors:** Kaitlin Kimmel, Michael Clark, David Tilman

**Affiliations:** ^1^ Department of Earth and Planetary Sciences Johns Hopkins University Baltimore Maryland USA; ^2^ Nuffield Department of Population Health University of Oxford Oxford UK; ^3^ Oxford Martin School University of Oxford Oxford UK; ^4^ Department of Zoology University of Oxford Oxford UK; ^5^ Interdisciplinary Centre for Conservation Science University of Oxford Oxford UK; ^6^ Ecology, Evolution and Behavior Department University of Minnesota St. Paul Minnesota USA; ^7^ Bren School of Environmental Science and Management University of California, Santa Barbara Santa Barbara California USA

**Keywords:** concurrent extinction drivers, extinction theory, long‐term extinction probability, probabilistic framework, synergistic effects, efectos sinérgicos, impulsores de la extinción concurrente, marco probabilístico, probabilidad de extinción a largo plazo, teoría de la extinción

## Abstract

Many species may face multiple distinct and persistent drivers of extinction risk, yet theoretical and empirical studies tend to focus on the single largest driver. This means that existing approaches potentially underestimate and mischaracterize future risks to biodiversity. We synthesized existing knowledge on how multiple drivers of extinction can interact to influence a species’ overall extinction probability in a probabilistic model of extinction risk that incorporated the impacts of multiple drivers of extinction risk, their interactions, and their accumulative effects through time. We then used this model framework to explore how different threats, interactions between them, and time trends may affect a species’ overall extinction probability. Multiple small threats together had potential to pose a large cumulative extinction risk; for example, 10 individual threats posed a 1% extinction risk each and cumulatively posed a 9.7% total extinction risk. Interactions among drivers resulted in escalated risk in some cases, and persistent threats with a small (1%) extinction risk each decade ultimately posed large extinction risk over 100 (9.6% total extinction risk) to 200 years (18.2% total extinction risk). By estimating long‐term extinction risk posed by several different factors and their interactions, this approach provides a framework to identify drivers of extinction risk that could be proactively targeted to help prevent species currently of least concern from becoming threatened with extinction.

## Resumen

Muchas especies pueden enfrentarse a múltiples impulsores distintivos y persistentes del riesgo de extinción, aunque los estudios teóricos y empíricos tienden a enfocarse en el impulsor más relevante. Esto significa que las estrategias existentes tienen el potencial de subestimar y caracterizar erróneamente los riesgos para la biodiversidad en el futuro. Sintetizamos el conocimiento existente sobre cómo los múltiples impulsores de la extinción pueden interactuar para influir sobre la probabilidad general de extinción de una especie en un modelo probabilístico del riesgo de extinción, el cual incorporó los impactos de los múltiples impulsores del riesgo de extinción, sus interacciones y sus efectos acumulativos a través del tiempo. Después usamos este modelo para explorar cómo las diferentes amenazas, las interacciones entre ellas y las tendencias temporales pueden afectar la probabilidad general de extinción de una especie. El conjunto de múltiples amenazas pequeñas tuvo el potencial de representar un gran riesgo de extinción acumulativo; por ejemplo, cada una de diez amenazas individuales representó 1% de riesgo de extinción, y acumuladas representaron un riesgo total de extinción de 9.7%. Las interacciones entre los impulsores resultaron en un riesgo escalado en algunos casos, y las amenazas persistentes con un riesgo pequeño (1%) de extinción durante cada década al final representaron un gran riesgo de extinción después de 100 (9.6% del riesgo total de extinción) y 200 años (18.2% del riesgo total de extinción). Mediante la estimación del riesgo de extinción a largo plazo que presentan los diferentes factores y sus interacciones, esta estrategia proporciona un marco para identificar los impulsores del riesgo de extinción que podrían focalizarse proactivamente para ayudar a prevenir que las especies que actualmente están en menor riesgo se conviertan en especies amenazadas.

## INTRODUCTION

Human activities are driving biodiversity loss as indicated by changes in International Union for the Conservation of Nature (IUCN) Red List Status (Williams et al., 2021), biodiversity intactness index (Newbold et al., 2015), community‐weighted biomass (Martin et al., 2019), and within‐species population abundances (WWF, 2020). A recent Intergovernmental Science‐Policy Platform on Biodiversity and Ecosystem Services report on biodiversity suggests that a million species are now threatened with extinction (IPBES, 2019). Continued biodiversity declines would mean that the biodiversity targets being discussed in the Post‐2020 Global Biodiversity Framework will be missed in decades unless rapid and ambitious actions to reduce human impacts on biodiversity are implemented.

Most species are affected by multiple human activities; over 80% of species on the IUCN Red List identified is affected by more than 1 anthropogenic activity (hereafter driver) (Maxwell et al., 2016). Human‐caused drivers of extinction include habitat destruction (Laurance et al., 2011; Tilman et al., 1994) and fragmentation (Haddad et al., 2015; Hanski & Ovaskainen, 2000); multiple aspects of climate change (Enright et al., 2015; McLaughlin et al., 2002; Urban, 2015); water and air pollution (Hayes et al., 2010); invasive species (Catford et al., 2018; Doherty et al., 2016); altered trophic interactions (Fortuna & Bascompte, 2006; Letnic et al., 2009; Schleuning et al., 2016); novel diseases (McCallum, 2012; McCallum et al., 2009); and mortality from hunting and harvesting (Burgess et al., 2017; Laurance et al., 2009; Ripple et al., 2016). Extinction drivers can have multiple mechanistic impacts on a species. Habitat destruction and fragmentation decrease habitat area; create small, isolated populations subject to deterministic and stochastic extinctions (Haddad et al., 2015; Rybicki & Hanski, 2013; Tilman et al., 1994); cause selective loss of some food web species, especially larger bodied herbivores and predators; and create novel environmental conditions at habitat edges (Laurance et al., 2006). Roadways destroy habitat, provide access for further destruction, and persistently elevate animal mortality in their vicinity (Fischer et al., 2018; Laurance et al., 2009). Climate change can reduce habitat quality by affecting temperature, precipitation, and humidity, which may place a species outside its range of physiological tolerance (Enright et al., 2015; McLaughlin et al., 2002; Thuiller et al., 2005; Urban, 2015) or may alter its competitive ability. Although any of the above mechanisms might directly drive a species to extinction, a persistent suite of such factors, each of which might seem on its own to be a minor risk, could in unison progressively decrease its population size (Brook et al., 2008); lower its net per capita rate of recovery after natural disturbances; and lead to greater oscillations in its population size, all of which would increase its extinction risk (Lande, 1988; Frankham, 2005; Hung et al., 2014).

Distinct drivers of extinction might also interact in ways that magnify or reduce the total extinction risk they pose. For example, as climate change occurs, habitat loss and fragmentation could prevent a species from migrating to suitable regions and thus could greatly increase its likelihood of extinction (Mantyka‐Pringle et al., 2015). Habitat fragmentation can concentrate prey species and provide easier access for hunters and poachers or natural predators, therefore increasing the extinction risk more than expected for an additive effect of the drivers as observed singly (Romero‐Muñoz et al., 2019). For example, the decline in crested terns (*Thalasseus bergii*) was driven synergistically by human harvest of eggs and typhoons (Chen et al., 2015). Terns that needed to relocate their nests after human harvest of eggs tended to make new nests in sites more prone to typhoons. Thus, the impact of these 2 drivers together was greater than expected based on their individual effects. There are other examples of 2 drivers acting together to make extinction risk greater than their independent effects (Didham et al., 2007; Laurance & Useche, 2009; Doherty et al., 2015), but there is still debate as to how prevalent these synergistic (or greater than additive) negative effects are (e.g., Leuzinger et al., 2011). However, understanding the impact of these potentially greater‐than‐expected interactions is essential for predicting extinction risk because such interactions may be a realistic scenario for some species. If, as current data suggest, most species are threatened by multiple interacting drivers of extinction and an increasing number of species will be affected by any single driver of extinction, a formal theory of the resultant extinction risk could provide insight and be a useful tool for conservation of biodiversity.

Despite that most species are affected by multiple human drivers, ecological theory aiming to quantify the extinction risk of a species has, to date, mainly focused on the link between a single driver and a species’ extinction risk. Although such studies provide valuable predictions about how different drivers may influence a species, they rarely address the accumulating effects of multiple concurrent and interacting drivers on extinction risk (but see Fortuna & Bascompte [2006] and De Laender [2018]). The multiple different and not directly comparable methods used to model extinction risk make it difficult to account for how a second or third driver, as well as the interactions between them, might affect a species’ likelihood of extinction. For example, much of the theory behind habitat loss and fragmentation relies on Levin's‐type metapopulation models (e.g., Tilman et al., 1994; Moilanen & Hanski, 1995), whereas much of the theory relating to small population sizes relates to demographic stochasticity and to the loss of genetic diversity of a species (e.g., Lande, 1988; Frankham, 2005). These theoretical approaches provide fundamental insights into major causes of extinction, but, as currently formulated, their fundamental focus remains on drivers considered singly. They are not designed to quantify the future extinction risk posed by a suite of simultaneous and interacting drivers. The existence of multiple persistent and simultaneous drivers of extinction risk suggests that a focus on a single driver could mischaracterize a species’ overall extinction risk.

We incorporated existing knowledge from ecological theory of the dynamics of drivers of extinction risk into a theoretical framework to investigate and illustrate how multiple simultaneous and persistent drivers of extinction risk and their interactions can jointly determine the overall and long‐term extinction risk of a species. We did this by developing a mathematical framework that incorporates and synthesizes knowledge from ecological theory to formally account for the cumulative impact of multiple drivers on extinction and for interactions between drivers that may increase or decrease a species’ risk of extinction. This perspective may be especially important for identifying species that may be on a path toward extinction because of multiple, small drivers (i.e., drivers that pose low risk of extinction on their own) that have not yet reduced population sizes sufficiently or so rapidly such that the species is deemed threatened with extinction.

## METHODS

To evaluate the combined and interactive effects of multiple drivers of extinction risk that are persistent through time, we built on the probabilistic framework of Gotelli (2008). Gotelli (2008) focused on metapopulation dynamics, identifying how the extinction risk of a given species varies through time or based on the number of populations in a species’ range. This framework does not include multiple simultaneous drivers of extinction, the potential interactions between them, or their cumulative impact on the extinction risk of a given species. We therefore expanded on this approach by incorporating these aspects of extinction risk in our mathematical framework.

The full equation, whereby the probability of extinction for a species (*P*_ex_), caused by *d* different drivers of extinction risk and by the interactions among these drivers is

(1) $$\;P_{\mathrm{ex}}={\left(1-\;\left(\prod_{i\;=\;1}^{d}\left(1-E_{i}\right)\;\right)\times {\left(\prod_{i\;=\;1,\;j\;>\;i}^{d}\left(1-X_{ij}\right)\right)}^{\tau }\right)}^{n}\;,$$

where *P*_ex_ for a population due to persistent driver *i* during a time interval of length *t* is *E_i_*. There are a total of *d* such drivers, with *i* = 1, 2, … *d*. The species occupies a total of *n* different sites, and the cumulative probability of extinction is determined for a total of *τ* time intervals, each of length *t*. Thus, *τ* = actual time/*t*. Moreover, *X_ij_* is the magnitude of the extinction risk created by an interaction between drivers *i* and *j*, and the *n* different sites are ecologically or geographically isolated or both but otherwise similar. The notation ∏ indicates the product of all terms. The *X_ij_* is necessarily bounded. When there is no interaction between the 2 drivers, *X_ij_* = 0. When the 2 drivers interact to increase the extinction probability, 0 < *X_ij_* < 1. When 2 drivers decrease the extinction probabilities over the multiplicative case, *X_ij_* is negative and bounded with $\frac{1}{E_{i}E_{j}}\left[1-\frac{1}{\left(1-E_{i}\right)\left(1-E_{j}\right)}\right]$ < *X_ij_* < 0.

More complicated scenarios not presented in the simplified version of the full model shown above were also considered. These are discussed below, and details are in Appendix S1.

### Extinction probability from multiple drivers

If there are 2 independent and noninteracting drivers of extinction risk, *E*_1_ and *E*_2_, then the chance of surviving both is $\left(1-E_{1}\right)\times \left(1-E_{2}\right)$. The probability of going extinct because of these 2 drivers during this period would then be $P_{\mathrm{ex}}=1-\left[\left(1-E_{1}\right)\left(1-E_{2}\right)\right]$. This simple equation can be generalized to any number of *d* distinct drivers of extinction risk, giving the cumulative extinction probability as

(2) $$\;P_{\mathrm{ex}}=1-\prod_{i\;=\;1}^{d}\left(1-E_{i}\right).$$

The probability of surviving for a time interval of *t* when faced with *d* distinct drivers of extinction is the product of all the individual survival probabilities (1 – *E_i_*) because we assumed independence between drivers. The total survival probability is subtracted from 1 to obtain the extinction probability per period for a species experiencing *d* different drivers.

### Extinction probability through time

As did Gotelli (2008), we accounted for cumulative extinction probability through time because many extinction drivers occur continuously or otherwise persist year after year. Again, *E_i_* is the extinction probability for a species for a period of length *t* (e.g., for extinction probability 0.01 per decade, *t* is a decade). To predict the extinction probability of a single driver for a longer period (e.g., after 10 decades), we used a metric of the number of periods, *τ*. The cumulative extinction probability with a single driver is

(3) $$P_{\mathrm{ex}}=1-{\left(1-E_{i}\right)}^{\tau }.$$

This probability of surviving through *τ* periods assumes that the probability of surviving each period is constant and independent of previous periods. Cumulative survival probability through time is estimated by raising the survival rate to the relevant number of periods. With *d* independent drivers of extinction each acting across all *τ* periods, the cumulative extinction probability is

(4) $$P_{\mathrm{ex}}=1-\prod_{i\;=\;1}^{d}{\left(1-E_{i}\right)}^{\tau }.$$

### Extinction probability from changing extinction probabilities through time

The *E_i_* might increase through time as climate change continues, as long‐lived pollutants accumulate, as more habitat is destroyed, and so forth (Brook et al., 2008). Thus, the impact of any 1 driver on extinction risk may increase if species are exposed to harsher conditions through time. Conversely, conservation efforts could progressively decrease the impact of any 1 driver on a species’ extinction probability.

An addition to the general framework is to account for the changing extinction risk of any 1 driver through time. This is done by having a new *E_i,t_* at each period so the cumulative probability over time at 1 location is

(5) $$P_{\mathrm{ex}}=1-\prod_{t\;=\;1}^{\tau }\prod_{i\;=\;1}^{d}\left(1-E_{i,t}\right).$$

The total extinction risk (*P*_ex_) differs based on the number of periods *τ* and on the risk posed by an individual driver, *i*. If a driver did not have an impact until a certain threshold (e.g., Clark et al., 2018), *E_i,t_* would be 0 until the period where this threshold is reached.

### Extinction probability from interacting drivers

To account for interactions between 2 drivers, we added *X_ij_*, which results in

(6) $$P_{\mathrm{ex}}=1-\left(\prod_{i\;=\;1}^{d}\left(1-E_{i}\right)\right)\times \left(\prod_{i\;=\;1,\;j\;>\;i}^{d}\left(1-X_{ij}\right)\right),$$

where *X_ij_* is necessarily bounded such that species cannot face more than a 100% chance or less than a 0% chance of extinction or persistence. Further, *X_ij_* only accounts for pairwise interactions, but there may be higher order interactions (i.e., among >3 drivers) as well.

### Extinction probability in multiple sites

Because of habitat fragmentation, many species are now distributed as spatially separated, independent populations across their geographic range. For example, a once contiguous population may now be separated into many smaller isolated populations. Following Gotelli (2008), we extended the model so populations would be located in multiple sites because for a species to go extinct, all of its independent populations must go extinct. We defined independent populations as those that with no movement between them. The *E_i_* values must reflect the actual status of these populations and would likely be larger than the *E_i_* value for an otherwise identical but contiguous population. For a species in *n* distinct but otherwise identical sites, the probability of extinction across its full geographic range is

(7) $$P_{\mathrm{ex}}={\left(1-\prod_{i\;=\;1}^{d}{\left(1-E_{i}\right)}^{\tau }\right)}^{n}.$$

We assumed that the magnitude and number of drivers is equal at all sites, but this may not be the case for many species. To account for the difference in the drivers and magnitude of their impacts, one would simply take the product of all the cumulative extinction probabilities from each site instead of raising the cumulative extinction probability to the *n*.

### Correlations with IUCN Red List data

We collected data from IUCN Red List on the number of distinct threats experienced by each species. We then correlated the number of distinct threats against the species’ report IUCN Red List Status to investigate whether there was a correlation between the number of threats a species experienced and its red‐list status.

## RESULTS

### Extinction probability from multiple drivers

**FIGURE 1:**
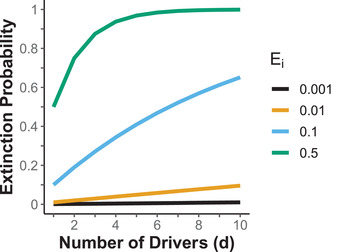
Impact of multiple independent threats on cumulative species extinction probability when each driver imposes the same extinction probability (*E_i_*)

**FIGURE 2:**
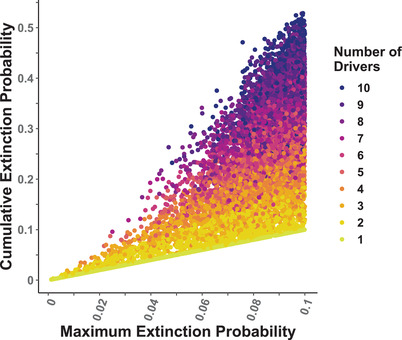
Cumulative species extinction probability within 1 period (*τ*) for 1–10 drivers versus the maximum extinction probability of each single driver. Results are from a simulation for which from 1 to 10 independent drivers were selected and extinction probability of these drivers was randomly assigned a value of 0.001 to 0.1 a uniform distribution

Multiple drivers of extinction posed a cumulative risk to extinction. For example, when we randomly chose multiple times from a uniform distribution bounded between 0.001 and 0.100 each of 10 *E_i_* values, the total extinction probability when each individual driver posed a low probability of extinction was approximately (but slightly less than) the sum of the probabilities of each driver, assuming independence between drivers. Thus, when all *E_i_* were small, *P*_ex_ ∼ *E*_1_ + *E*_2_ + … + *E_d_* (Equation 2 & Figure 1). For example, when a population had an extinction probability of 1% (*E_i_* = 0.01) for each of 10 different drivers, the overall extinction probability was ∼9.7%, an approximately additive effect. Therefore, multiple drivers that had small individual effects can, in combination, posed substantial threats for a species. Clearly, a species affected by multiple drivers had a higher cumulative extinction probability than species affected by a single large driver (Figure 2). A similar trend was found with IUCN Red List data, which indicated that terrestrial vertebrates with more drivers of extinction on average experienced a higher risk of extinction than vertebrates with below average number of drivers (Appendix S2). Additionally, the difference between extinction risk posed by the single largest driver and the cumulative extinction risk increased as the number of drivers affecting the species increased.

### Extinction probability through time

**FIGURE 3:**
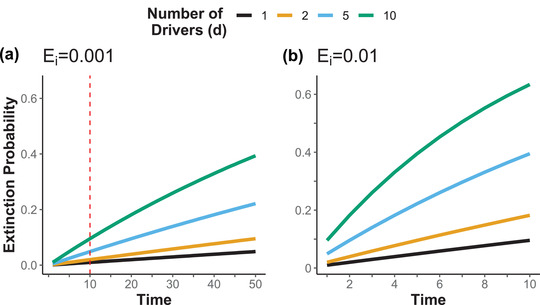
Species extinction probability through time for different numbers of drivers, where each driver poses an extinction probability (*E_i_*) of (a) 0.1% or (b) 1% (dashed line, time step where graph ends; timescales on graphs differ)

In the long term, the several unmitigated, small but persistent drivers cumulatively posed a large extinction risk to a species because the cumulative probability of extinction increased through time (Equations 3 & 4). When considering only 1 driver with a relatively small extinction probability, extinction probability increased almost linearly through time (Figure 3, black lines). For example, based on Equation 3, at the end of 10 periods, a species with a 0.1% chance of going extinct from 1 driver would have an extinction risk of 0.996%, which is about 10 times greater than after only 1 period (Figure 3a). Although the cumulative impact of time and multiple independent drivers was necessarily less than purely additive impact (Equation 4 & Figure 3), a species affected by many small drivers for many time steps had a much higher chance of extinction than a species affected by just a few drivers (e.g., the 10‐driver curve saturated before the 5‐driver curve in Figure 3). With multiple small persistent drivers of extinction, species were likely to go extinct much faster (in evolutionary time) than has been predicted based on background extinction rates (Pimm et al., 2014; Ceballos et al., 2015).

### Extinction probability from changing extinction probabilities through time

**FIGURE 4:**
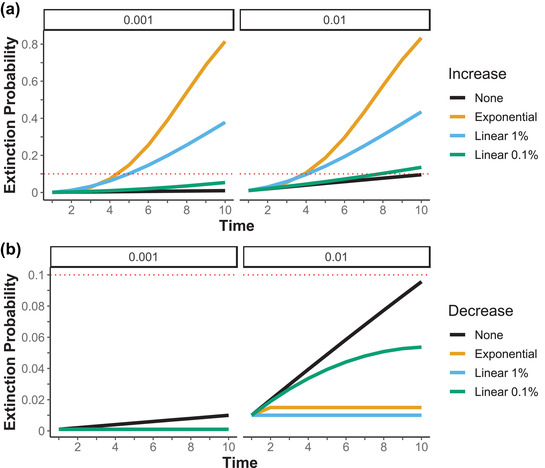
Extinction probability through time when the severity of a single driver (a) increases (initial extinction risk 0.1%) or (b) decreases (initial extinction risk 1.0%) through time relative to the magnitude of the increase or decrease (red dashed lines, same magnitude of extinction probability of 0.10 or 10%)

When extinction probability increased with each time step, such as from continuing climate change or continuing habitat loss, the cumulative extinction probability changed from being an approximately linear increase through time to being a more than linear increase (Figure 4a). The initial extinction risk mattered little when extinction probability increased substantially between time steps. When extinction probability decreased with each time step, such as from conservation efforts, the cumulative extinction probability depended on the initial risk and the rate of reduction (Figure 4b).

### Extinction probability from interacting drivers

**FIGURE 5:**
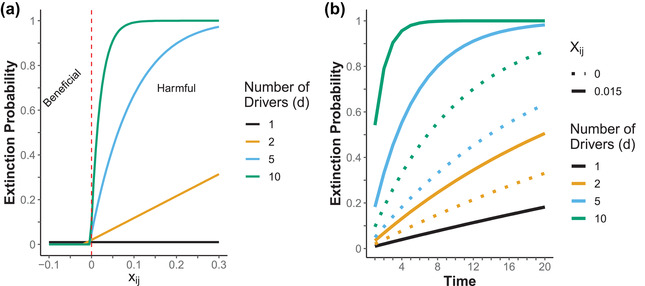
Cumulative extinction probability relative to (a) interaction between drivers (*X_ij_*, magnitude of extinction risk created by interaction between drivers *i* and *j*) and (b) persistence of these drivers and their interactions through time (driver of extinction risk 0.01 for all drivers)

Harmful interactions greatly increased extinction risk, just as beneficial interactions rapidly decreased extinction risk (Equation 6 & Figure 5a). The magnitude of this change depended on the strength of the interaction and the number of drivers with interactions (Figure 5b). For example, 5 drivers with a small interaction between each combination of drivers were more harmful than 10 drivers with no interactions (Figure 5b).

### Extinction probability in multiple sites

**FIGURE 6:**
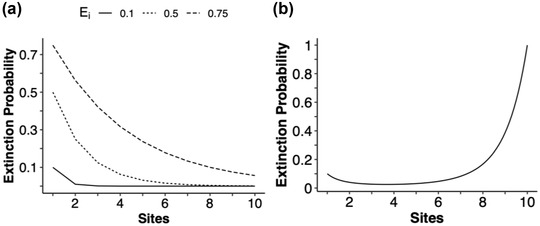
Extinction probability at a single point in time relative to the number of sites in a species’ range: (a) extinction risk (*E_i_*) is independent of the number of sites in a species’ habitat range and (b) *E_i_* scales with the number of sites (*n*), such that *E_i_* = 0.1 × *n* because each additional site in a species’ range could mean that any given site has fewer individuals and is thus more prone to extinction

Species with populations in many independent sites had a lower probability of extinction, all else being equal (e.g., all had the same *E_i_*), than species that had only 1 or a few similarly sized local populations (Figure 6). This is not to say that fragmented populations fared better than large populations. Rather, a species with populations in multiple independent locations fared better than a species that had fewer such populations, given that sites were independent of each other and all else, including abundances of the species, being similar across the sites. Extinction increased as habitat was destroyed and turned into habitat remnants.

Our model showed that habitat fragmentation can greatly increase a species’ extinction risk. For a species that experienced habitat loss that resulted in *n* distinct fragmented habitat sites, each site had a population size much less 1/*n* of the size of the former fully intact population. This resulted in an extinction risk much greater than *n* times the original extinction risk when the habitat was fully intact. Thus, species with 1 large population that was split into many smaller isolated populations had an elevated extinction risk (Figure 6b).

## DISCUSSION

Our mathematical framework showed that species unaffected by a single major extinction risk may nonetheless have high extinction probabilities if they are affected by multiple, persistent, small drivers and harmful interactions between these drivers. Therefore, existing theoretical approaches that focus on a single driver of extinction risk might mischaracterize the total extinction risk for a species. This is supported by numerous field‐based surveys that show that the cumulative effect on biodiversity of multiple drivers can be more (e.g., Chen et al, 2015), less (e.g., Reich, 2009), or equivalent to the additive impact of independent drivers (e.g., Mora et al, 2007). It is also supported by recent analyses in which field‐based observations were used to construct a framework to assess extinction risk from multiple simultaneous human activities (Lum et al., 2020; Zhang & Vincent, 2019). Furthermore, our model results suggest that species not currently identified as threatened with extinction but that experience multiple, small, and persistent risks may experience a large long‐term extinction risk over long periods because the extinction risk from many low‐level persistent drivers are approximately additive and the risks increase through time. This is also supported by IUCN data, which show that species experiencing more threats have, on average, a larger risk of extinction (Appendix 2), and imply that reducing the largest threat to a species may not substantially reduce total extinction risk if it faces many small risks. Thus, it is important to work toward updating current predictive models, which usefully identify species facing the greatest extinction risk and in need of conservation, to more fully account for the cumulative extinction risk that results from multiple persistent drivers and their interactions through time.

Empirical studies of threatened species often report multiple causes of population declines, thus suggesting that our approach may be needed to understand their long‐term susceptibility to extinction. Insect pollinators and amphibians provide 2 taxonomically distinct examples of organisms experiencing many potentially small and persistent threats to extinction. Multiple species in each of these taxa are threatened by at least 10 unique drivers (Wake & Vredenburg, 2008; Thogmartin et al., 2017; Wagner et al., 2021). Amphibian species are threatened by novel diseases (Daszak et al., 2003; Martel et al., 2013); habitat loss (Lehtinen et al., 2003); habitat fragmentation (Becker et al., 2007); increasing temperatures (Ficetola & Maiorano, 2016); changing precipitation patterns (Ficetola & Maiorano, 2016); pollutants, such as triclosan (Regnault et al., 2018) and atrazine (Hayes et al., 2010); overexploitation (Stuart et al., 2004); and invasive species (Blaustein & Kiesecker, 2015). Likewise, insect pollinators are threatened by pesticides (Goulson et al., 2015); habitat loss (Brown & Paxton, 2009); reduced food sources (Zaya et al., 2017); climate‐change‐induced phenology shifts in their plant host species (Settele et al., 2016); decoupling of plant and pollinator ranges (Settele et al., 2016); invasive predators (Vanbergen et al., 2018); invasive competitors (Vanbergen et al., 2018); parasites (Vanbergen et al., 2018); diseases (Cameron et al., 2011); pollutants (Evans et al., 2018); and altered trophic interactions (Vanbergen et al., 2018). It seems that our framework would be applicable to species such as these and those less well studied that may be facing lower level multiple threats.

Empirical studies show that it is difficult to accurately quantify extinction probability over long timescales because of the quantity of information needed and because future conditions may not have a historical analog or may be unknown (Fieberg & Ellner, 2000; Coulson et al., 2001). It may also be difficult to disentangle the separate effects of multiple drivers on a species through observational studies. Thus, it is necessary to continue to gather this type of information from experiments in which many of these drivers are manipulated separately and jointly and from observational studies of species that are currently not threatened with extinction to determine what drivers could pose risk in the future. Further, our framework may be useful to fill in gaps where information is not available on the risk from specific drivers. For example, researchers could assume some small nonzero chance of extinction by drivers for which probability cannot be quantified empirically. This would allow them to get an estimate from which to work.

Within our framework, pairwise interactions between drivers might increase or reduce extinction probability, but there is still little consensus empirically as to the direction of such interactions. There is evidence for more than additive interactive increases in risks (e.g., Mora et al., 2007; Mantyka‐Pringle et al., 2012; Chen et al., 2015); less than additive effects of interactions (e.g. Reich, 2009); and no interactions between drivers (e.g. Mora et al., 2007; Yue et al., 2020). Metanalyses also report evidence for all 3 possible types of interactions among drivers (Darling & Côté, 2008; Piggott et al., 2015). Clearly, further work is needed on this issue.

With our model, we assumed that separate populations of a species are independent. Metapopulation dynamics, though, are based on the assumption that some movement between populations occurs (Hanski, 1998; Hanski & Ovaskainen, 2002). Sites connected by movement of individuals could provide a rescue effect. Individuals can recolonize sites after a local extinction and restart the local population or reduce extinction risk by providing more individuals or more genetic variation (Hanski, 1999). However, nonindependent sites could increase extinction probability if migration, such as by diseased individuals, were to increase mortality sufficiently.

Further, experimental evidence shows that a suite of fragmented habitats can lose species much faster than a single habitat of the same total size (Haddad et al., 2017). Thus, in small habitat patches, extinction probabilities could be higher due to negative impacts of increased edge area (Laurance et al., 2006), human access (Romero‐Muñoz et al., 2019), and small population sizes (Mace et al., 2008). To determine how common these metapopulation effects are and how likely they are to reduce extinction risk, information will be needed on how connected different fragments are, what species move between these habitats, and how such movement impacts extinction risk (Hanski, 1998). Because of how the spatial patterning of populations within a species’ habitat range can increase, decrease, or not affect a species’ extinction risk and how this spatial pattern can influence on‐the‐ground conservation planning, our approach would be best used to derive general trends in extinction risk for a species or a set of species. Species identified as at high risk of extinction could then be examined in more depth with an approach that accounts for how multiple drivers of extinction interact with the spatial patterns of a species’ habitat and should therefore be pursued. Species traits could also mitigate or amplify extinction risk from a single driver. For example, species with short generation length are more likely to experience significant die off (i.e., >50% of a population is lost) in a given year than are species with longer generations (Reed et al., 2002).

Our framework provides an additional way to view extinction risk and is complementary to the existing wealth of theory and empirical knowledge about the causes of extinction. Our approach quantifies how extinction risk could be affected by multiple persistent risk factors, their interactions, spatially independent populations, and time. We suggest that this perspective may be helpful in forecasting the long‐term implication of seemingly small and low‐risk drivers of extinction, which can be further paired with approaches that assess how limited conservation resources can be effectively allocated to reduce extinction risk from multiple simultaneous extinction drivers (Moore et al., 2021). In total, our model raises 3 important points. First, multiple small threats may, in total, pose significant extinction risk to a given species. Second, harmful interactions between risks may do the same. Third, if 1 or more threats are persistent, even a seemingly small risk of extinction per decade could have devastating impacts after a century or 2.

## Supporting information

Figure S1. Visual diagram of the equations developed in the manuscript

Figure S2. Correlation between number of drivers a species experiences and the average IUCN Red List status of species
