# Supplementary material for: Impact of multiple small and persistent threats on extinction risk
Source: Conserv Biol. 2022 May 5;36(5):e13901. doi: 10.1111/cobi.13901 (PMC9790556; doi:10.1111/cobi.13901)
Supplement: Supplementary file 1 — Figure S1. Visual diagram of the equations developed in the manuscript [file COBI-36-0-s001.docx]

**Appendix S1.**

Figure S1. Visual diagram of the equations developed in the manuscript. The diagram builds from extinction risk from a single driver at a single point in time (left side), to cumulative extinction risk from multiple interacting drivers through time, with the magnitude of individual drivers changing, assuming the species is split into multiple independent sub-populations (as in equation 1 in the manuscript). Corresponding equations in the manuscript are indicated with **bold text**, and additional mechanisms added between equations are indicated by arrows and text. All vector art is from the Noun Project, and includes: Cutting Down a Tree by Gan Khoon Lay; Pesticides by Yu Luck; Time by BomSymbols; and Line Graph by Thomas Le Bas.
