# Supplementary material for: Impact of multiple small and persistent threats on extinction risk
Source: Conserv Biol. 2022 May 5;36(5):e13901. doi: 10.1111/cobi.13901 (PMC9790556; doi:10.1111/cobi.13901)
Supplement: Supplementary file 2 — Figure S2. Correlation between number of drivers a species experiences and the average IUCN Red List status of species [file COBI-36-0-s002.docx]

**Appendix S2.**

*
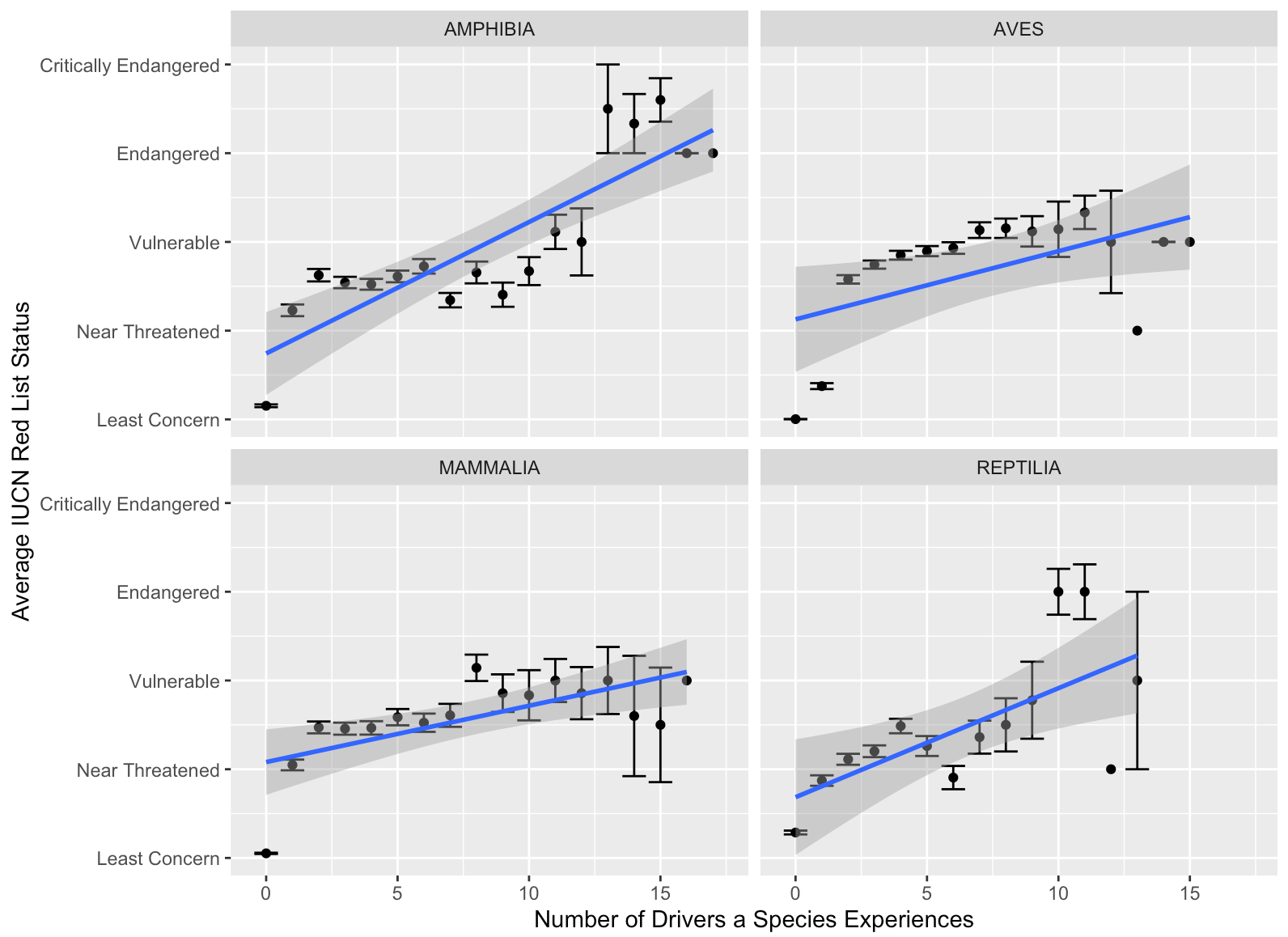
*

Figure S2. Correlation between number of drivers a species experiences and the average IUCN Red List status of species. Analyses shown are for terrestrial vertebrates, with separate panels for Amphibia, Aves, Mammalia, and Reptilia. To plot the data, the IUCN Red List Status was converted to a 1-5 numeric scale, whereby: Least Concern = 1; Near Threatened = 2; Vulnerable = 3; Endangered = 4; and Critically Endangered = 5. Points indicate mean estimate, and error bars indicate +- 1 s.e.m.
